# American Medical Association

**Published:** 1875-04-01

**Authors:** Wm. B. Atkinson

**Affiliations:** Permanent Secretary; Philadelphia, 1400 Pine St.


### Miscellaneous.

### AMERICAN MEDICAL ASSOCIATION.

THE Twenty-sixth Annual Session will be held in the city of Louisville, Ky., on Tuesday, May 4th, 1875, at 11 A. M.

"The Delegates shall receive their appointment from permanently organized State Medical Societies, and such County and District Medical Societies as are recognized by representation in their respective State Societies, and from the Medical Department of the Army and Navy of the United States."

"Each State, County and District Medical Society entitled to representation shall have the privilege of sending to the Association one delegate for every ten of its regular resident members, and one for every additional fraction of more than half that number: *Provided*, however, that the number of delegates for any particular State, territory, county, city or town shall not exceed the ratio of one in ten of the resident physicians who may have signed the Code of Ethics of the Association."

"The Chairmen of the several sections shall prepare and read in the general sessions of the Association, papers on the advances and discoveries of the past year in the branches of science included in their respective sections. * * *"—*By-Laws*, *Art. II.*, *Sec.* 4.

SECTIONS.

Practice of Medicine, Materia Medica, and Physiology: Dr. Austin Flint, Sr., New York, N. Y., *Chairman*; Dr. J. K. Bartlett, Milwaukee, Wis., *Secretary*.

Special Committees appointed to report to this Section:

On Meteorological Observations: Dr. J. M. Toner, D. C., *Chairman;* Dr. J. J. Woodward, U. S. A.; Dr. E. Lloyd Howard, Md.

On Clinical Observations: Dr. N. S. Davis, Ill., *Chairman;* Dr. H. A. Johnson, Ill.; Dr. J. B. Johnson, Mo.

Obstetrics and Diseases of Women and Children: Dr. W. H. Byford, Chicago, Ill., *Chairman;* Dr. S. C. Busey, Washington, D. C., *Secretary.*

Special Committees to prepare business for this Section:

Dr. M. A. Pallen, N. Y., *Chairman;* Dr. L. F. Warner, Mass.; Dr. J. K. Bartlett, Wis.

Committees appointed by the above:

On Unusual Fœtal Presentation: Dr. J. A. Octerloney, Ky.

On Retroversion of the Uterus in the first five months of Pregnancy: Dr. Heaton, Mich.

On the Connection of the Hepatic Circulation with Uterine Hyperæmias, Fluxions, Congestions, and Inflammations: Dr. L. F. Warner, Mass.

On the Relation of Menstruation during Lactation: Dr. S. C. Busey, D. C.

Surgery and Anatomy: Dr. E. M. Moore, Rochester, N. Y., *Chairman;* Dr. T. S. Latimer, Baltimore, Md., *Secretary.*

Committee to report to this Section:

On the Treatment of Fractures: Dr. Lewis Sayre, New York, *Chairman.*

Medical Jurisprudence, Chemistry, and Psychology: Dr. Jerome Cochran, Mobile, Ala., *Chairman;* Dr. G. A. Moses, St. Louis, Mo., *Secretary.*

State Medicine and Public Hygiene: Dr. H. I. Bowditch, Boston, Mass., *Chairman;* Dr. H. B. Baker, Lansing, Mich., *Secretary.*

Committees to report to this Section:

On Ventilation of Dwellings, School Houses, and other Public Buildings: Dr. R. C. Kedzie, Mich., *Chairman;* Dr. A. B. Stuart, Minn.; Dr. R. J. O'Sullivan, N. Y.

On Form of Bill to Establish a National Department of Public Health at Washington: Dr. H. B. Baker, Mich., *Chairman;* Dr. H. A. Johnson, Ill.; Dr. J. M. Toner, D. C.

On what Legislative Action, if any, can be taken to Enforce by Law an Examination of all Persons who enter upon the Practice of Medicine and Surgery, by a State Board of Medical Examiners: Dr. Foster Pratt, Mich., *Chairman*; Dr. S. G. Armor, N. Y.; Dr. D. W. Yandell, Ky.

☞ "Papers appropriate to the several sections, in order to secure consideration and action, must be sent to the Secretary of the appropriate section at least one month before the meeting which is to act upon them. It shall be the duty of the Secretary to whom such papers are sent, to examine them with care, and, with the advice of the Chairman of his section, to determine the time and order of their presentation, and give due notice of the same. * * *"—*By-Laws, Art. II., Sec.* 5.

The following Committees are expected to report:

On Cultivation of the Cinchona Tree: Dr. L. J. Deal, Penn., *Chairman*.

On some Diseases Peculiar to Colorado: Dr. John Elsner, Col., *Chairman*.

On American as compared with Foreign Winter Cures: Dr. H. R. Storer, Mass., *Chairman*.

On Railroad Injuries: Dr. W. F. Peck, Iowa, *Chairman*.

On Proper Legislation to Prevent the Spread of Syphilis: Dr. S. D. Gross, Pa., *Chairman*.

On the Use of Pessaries: Dr. John Morris, Md., *Chairman*.

On Cystic Degeneration of the Kidneys: Dr. John A. Octerloney, Ky., *Chairman*.

On the Diseases of Minnesota and the Northwest: Dr. D. W. Hand, Minn., *Chairman*.

On Prize Essays: Dr. John Davies Jackson, Ky., *Chairman*.

On Necrology: Dr. S. C. Chew, Md., *Chairman*.

On Rank of Medical Department of the Army: Dr. J. M. Toner, D. C., *Chairman*.

On International Medical Association: Dr. J. M. Toner, D. C., *Chairman*.

On Memorial on Dr. Henry Miller, deceased: Dr. S. D. Gross, Pa., *Chairman*.

On Memorial on Dr. George Mendenhall, deceased: Dr. J. A. Murphy, Ohio, *Chairman*.

The following amendments to the Plan of Organization are to be acted upon:

By Dr. H. B. Baker, Michigan:

"The officers of the several Sections shall be nominated by the Section in and for which said officers are to serve."

By Dr. Adams Jewett, Ohio:

"The Permanent Members shall consist of all those who have served in the capacity of delegates, and of such other members as shall have received the appointment by unanimous vote, and of all others who, being members in good standing of any State or local medical society entitled to representation in this body, shall, after being vouched for by at least three members, be elected to membership by a vote of three-fourths of the delegates in attendance, and shall continue such so long as they remain in good standing in the body of which they were members when elected to membership in this Association, and comply with the requirements of its By-Laws."

☞ Secretaries of all State Medical Societies that have adopted the Code of Ethics are respectfully requested to forward to the undersigned a complete list of the officers, with their post-office addresses, of those County and District Medical Societies entitled to representation in their respective bodies. This is the only guide for the Committee of Arrangements in determining as to the reception of delegates.

It will also enable the Permanent Secretary to present a correct report of the medical organizations in fellowship with the Association.

WM. B. ATKINSON, M.D.,
*Permanent Secretary.*

PHILADELPHIA, 1400 Pine St.

